# How are agitated patients dealt with in internal medicine departments?

**DOI:** 10.1097/j.pbj.0000000000000260

**Published:** 2024-07-11

**Authors:** José António Ferraz-Gonçalves, Ana Sofia Silva, Joana Silva Reis, José Guilherme Assis, Maria Inês Matos, Paula Matias, Sérgio Alves

**Affiliations:** a Faculdade de Medicina da Universidade do Porto, Porto, Portugal; b Serviço de Medicina Interna, Unidade Local de Saúde Gaia e Espinho, Vila Nova de Gaia, Portugal; c Serviço de Oncologia Médica, Unidade Local de Saúde São João, Porto, Portugal; d Serviço de Medicina Interna, Unidade Local de Saúde de Trás-os-Montes e Alto Douro, Vila Real, Portugal; e Serviço de Medicina Interna, Unidade Local de Saúde São João, Porto, Portugal; f Serviço de Medicina Interna, Unidade Local de Saúde Nordeste, Bragança, Portugal

**Keywords:** agitation, physical restraints, verbal de-escalation, agitation assessment, agitation control

## Abstract

**Background:**

Studies on agitation in internal medicine departments are scarce, especially regarding how doctors and nurses act in these situations. The objective of this study was to clarify how agitation is dealt with in these departments.

**Methods:**

This prospective observational study was performed in the internal medicine departments of four Portuguese hospitals. The researchers at each hospital contacted the nursing team that identifies patients who were agitated in the previous shifts. The researcher reviewed these patients' files, recording the research protocol's parameters.

**Results:**

During the study period, 331 patients were observed; 177 (54%) were female, and the median age was 80 years (19–99). Episodes of agitation occurred in 69 patients (21%); of them, 44 (64%) were female, and the median age was 84 years (31–98). In the first episode of agitation, the doctor on duty was called in 49 times (71%). These doctors prescribed a new medication for the crisis in 30 cases (43%). After the crisis, the assistant doctor recorded the episode in the patient file in 41 cases (59%). According to the medical notes, after the acute phase, in only 21 patients (30%), there was an attempt to clarify the cause of agitation. The prescription after the crisis was regular medication in 32 cases (46%), rescue medication in 27 (39%), and physical restraint in 9 (13%), isolated or in various combinations.

**Conclusion:**

This study suggests that there is room to improve how agitated patients are managed in internal medicine departments.

## Introduction

Episodes of agitation occur frequently in inpatients in several settings, namely intensive care,^1^ in the postoperative period,^2^ internal medicine departments,^3^ geriatric departments,^4^ and palliative care.^5^ This phenomenon has not been evenly studied across all these kinds of departments. In internal medicine departments, agitation has not been so extensively studied as in, for example, intensive care. Nevertheless, admissions to internal medicine departments are incomparably more frequent than admissions to intensive care. Although there is the idea that agitation in internal medicine departments is relatively frequent, there are no numbers to confirm it in Portugal. Even internationally, studies on this subject are limited. Besides agitation prevalence, there are no data on how doctors and nurses act in these situations.

Psychomotor agitation is defined in the International Classification of Diseases-11 as “excessive motor activity, usually manifested by purposeless behaviors such as fidgeting, shifting, fiddling, inability to sit or stand still, and wringing of the hands.”^6^ Agitated patients may pull out venous catheters, nasogastric tubes, gastrostomy tubes, vesical catheters, and other devices. They may even attack health professionals and other patients. Even if aggression and violence are not defining features of agitation, if agitation progresses in severity, aggressive and violent behaviors may occur.^7^ Other definitions of agitation are reasonably consistent, but it remains a broad multifactorial syndrome with a lack of unequivocal agreement.^7^

Agitation may have several different causes, such as delirium, dementia, psychosis, and general medical conditions.^7^ Agitated patients are disruptive mainly at night, when most agitation episodes occur and when the number of nurses is lower. They may be a danger to themselves and others and require rapid intervention. However, doctors frequently disregard agitation; even when they do not, they often neglect its investigation and treatment.^8^

How health professionals deal with agitated patients in Portuguese internal medicine departments at each agitation episode, how doctors value agitation episodes, and, therefore, how they investigate their causes are unknown. This study aims to respond to these doubts. This study does not intend to determine the causes or risk factors of agitation by comparing the parameters of agitated patients with patients who were not agitated. The main objective of this study is to figure out how health professionals, mainly doctors, deal with agitated patients. The results of this study may raise awareness of possible failures in the way agitated patients are managed and, consequently, induce improvement in this crucial aspect of medical care.

## Methods

This prospective observational study was performed in five Portuguese hospitals. The main objective was to determine how agitated patients are managed within internal medicine departments. A secondary objective was determining the frequency of agitation episodes in internal medicine departments.

The inclusion criteria were patients older than 18 years, admitted to an internal medicine ward in one specific week (Monday to Sunday) at each hospital, and remaining for at least 24 hours in that ward. Patients were followed until death, hospital discharge, transference to another institution, or accomplishing one month of follow-up.

Agitation was defined as disruptive vocalizations, such as calling out or screaming; hallucinations; hypervigilance; aggressivity/combativity; wandering; noisemaking; uncooperative with care; repeated falls, unexplained by other reasons; pulling out catheters or venous accesses; or other disturbing behaviors.

Doctors or nurses identified agitated patients. One of the researchers at each hospital contacted the nursing team that identified patients with agitation in the previous shifts. After the assistant physician's visit, the researcher reviewed these patients' files, recording the research protocol's parameters. This process was repeated every day until discharge, death, or the end of the study (30 days after admission).

Besides the demographic data, the recorded data included the immediate action in the first episode of agitation, assessment, and control; data on the follow-up after the episode, including the record by the doctor who usually follows the patient, assessment treatment, and measures to control possible future crises; and the reasons for patients leaving the study, whether death, discharge, or end of the study (after one month of follow-up). The data recorded from patients without episodes of agitation included demographics, primary clinical data, and reasons for leaving the study.

Regarding statistics, descriptive methods were used. The χ^2^ test was used to compare nominal variables and the Mann–Whitney test for continuous variables. The level of significance was deemed to be .05. The 29.0 version of IBM SPSS statistical software was used to analyze the data.

Because the study was observational of the standard practice, without any intervention, therefore not involving any risk for patients, and the detailed data collected were from agitated patients without cognitive competence to give informed consent, the need for consent was waived according to article 17 of the Oviedo Convention.^9^ Patients could not be identified because their data were anonymized.

The study was submitted to and authorized by the ethics committee and the board of directors of each hospital.

## Results

During the study period, 331 patients were observed; 177 (54%) were female, and the median age was 80 years (19–99). Episodes of agitation occurred in 69 patients (21%); of them, 44 (64%) were female and the median age was 84 years (31–98). The difference between agitated and nonagitated patients concerning sex was not significant (*P*=.054; chi-square) but was significant concerning age (*P*=.002; Mann–Whitney). The leading cause of admission to the hospital was infection (Table 1).

**Table 1:** Cause of admission.

Disease	N	%
Infectious	140	42
Neurological	29	9
Respiratory	62	19
Cardiac	32	10
Hepatic	15	5
Diabetes mellitus	10	3
Miscellaneous	43	13
Total	331	100

In the first episode of agitation, the doctor on duty was called in 49 cases (71%). These doctors prescribed a new medication for the crisis in 30 cases (43%). The patients received the prescribed rescue medication in 30 cases (43%). In 29 cases (42%), physical restraints were used. Verbal de-escalation was tried in only 14 patients (20%) (Table 2).

**Table 2:** Initial response to the crises.

Intervention	N	%
Intervention of a doctor on call	49	71
Prescription of new medication	30	43
Physical restraint	29	42
Verbal de-escalation	14	20
Prescribed rescue medication	30	43

This table shows the initial reaction to the first crisis of agitation. In some patients, more than one intervention was made.

An antipsychotic alone was administered to 19 patients (28%); in 11 (16%), a benzodiazepine alone, and in 8 (12%), a combination of an antipsychotic and a benzodiazepine (Table 3). The antipsychotic most used was haloperidol in 19 patients (28%), followed by quetiapine, and the benzodiazepine more often given was diazepam in 9 patients (13%), followed by midazolam (Table 3). Morphine was administered to 8 patients (12%). With the interventions, 55 episodes (80%) were deemed controlled.

**Table 3:** Medication used the control of the first crisis.

Haloperidol	19	28%
Quetiapine	10	14%
Diazepam	9	13%
Midazolam	8	12%
Morphine	8	12%
Lorazepam	3	4%
Oxazepam	2	3%
Risperidone	2	3%
Alprazolam	1	1%
Bromazepam	1	1%
Chlorpromazine	1	1%
Olanzapine	1	1%
Other	14	20%
None	12	17%
Antipsychotic alone	19	28%
Benzodiazepine alone	11	16%
Antipsychotic/benzodiazepine	8	12%

The doctor on duty assessed blood oxygen saturation and vital signs in 55% of the agitated patients and capillary blood glucose in 39% (Table 4). The working hypotheses most often considered were the development of an acute condition and the possibility of patients having had recurrent episodes of agitation. Medication withdrawal was also considered in 10%, but medication toxicity was never hypothesized. In 33% of the cases, nothing was stated in the patient file.

**Table 4:** Considerations by the on-call doctor at the first episode.

	n	%
Basic assessment		
Peripheral oxygen saturation	38	55
Vital signs	38	55
Capillary blood glucose	27	39
Working hypotheses considered		
Acute condition	20	29
Previous episodes of agitation	13	19
Medication withdrawal	7	10
Urinary retention	3	4
Medication toxicity	0	0
Not stated	23	33

The first episode of agitation was isolated in 45 patients (65%), frequently followed by occasional episodes in 18 patients (26%) and by frequent episodes in 6 (9%).

After the crisis, the assistant doctor recorded the episode in the patient file in 41 cases (59%). After the acute phase, in only 21 patients (30%), there was an attempt to clarify the cause of agitation. Of the patients who were not investigated to determine a cause of agitation, 21 (30%) had a cause already known; in 9 (13%), the episode was isolated and not severe; and in 18 (26%), the reason was not apparent. The prescription after the crisis was regular medication in 32 cases (46%), rescue medication in 27 (39%), and physical restraint in 9 (13%), isolated or in various combinations.

When the doctor tried to discover the cause of the agitation, they considered several hypotheses, including infectious or other acute conditions, neurological or psychiatric disorders, kidney or liver impairment, and medication introduced or withdrawn (Table 5).

**Table 5:** Workup for the cause of agitation after the first episode.

Action	N	%
Search for an infectious focus	16	23
Previous cognitive impairment	15	22
Search for other acute conditions	11	16
Hydroelectrolytic alteration	9	13
Brain CAT or MRI	8	12
Previous agitation episodes	7	10
Previous psychiatric disease	7	10
Neurological abnormalities	7	10
Impairment of renal function	7	10
Impairment of liver function	7	10
Medication withdrawal	4	6
Medication introduced	4	6
Impairment of thyroid function	3	4
Nutritional abnormalities	3	4

This table shows what doctors looked for after the first episode of agitation to explain its cause.

CAT, computed axial tomography; MRI, magnetic resonance imaging.

The most frequent reason for leaving the study was discharge from the service, followed by death (Table 6). Although there were some differences in the reasons for leaving the study between patients with an agitation episode and those without, they were not statistically significant.

**Table 6:** Reasons for leaving the study by comparing patients with and without agitation.

Reason for leaving the study	Agitation				*P*
	No		Yes		
Discharge	184	70%	43	62%	.073
Death	39	15%	7	10%	
Transference to other facility	28	11%	4	6%	
End of the study	11	4%	7	10%	

## Discussion

This study explores the attitudes of physicians concerning agitation. There are many studies on the prevalence and causes of agitation^3,10,11^ and recommendations and guidelines on its management.^12,13^ However, it is difficult to find studies on how physicians act toward agitated patients.

Agitated patients often endanger other patients, the staff, and themselves. Therefore, a quick actuation is necessary. However, coercive methods should be avoided whenever possible. Verbal de-escalation is one of those methods. In this study, verbal de-escalation was tried in 20% of the agitated patients. A 2017 Cochrane revision concluded that, although accepted as good clinical practice, verbal de-escalation is not supported by evidence from randomized trials.^14^ Another systematic review published in the same year also concluded that de-escalating interventions might effectively lower aggressive behavior and the use of seclusion and restraints, but the level of evidence needs to be higher and more research is needed.^15^ Nevertheless, in guidelines, de-escalation is still recommended as a first-line intervention, mainly for patients with mild or moderate agitation who can make eye contact and engage in conversation.^12^

Physical/mechanical restraints were used in 42% of agitated patients (9% of all patients). In another study performed in Portugal on delirium in internal medicine wards, 53% of patients with delirium had physical restraints.^10^ At the international level, the prevalence of restraints in hospitals has been reported as between 0% and 100%.^16^ These differences are probably due to differences in the definition of restraints, setting (eg, emergency department, intensive care, and internal medicine wards), or institutional policies. Many studies include bed rails as physical/mechanical restraints,^17,18^ but this study does not. In Portugal, bed rails are usually used when patients are in bed for security reasons, mainly at night, and, therefore, cannot be classified as restraints. Guidelines from the American College of Emergency Physicians state that patients' restraints “should be considered when a careful assessment establishes that the patient is in danger to self or others by virtue of a medical or psychiatric condition and when verbal de-escalation is not successful” and adds, “When appropriate and safe, verbal de-escalation and standard treatment of underlying medical or psychiatric conditions should be attempted before restraints.”^13^ Therefore, restraints should not be used as a first-line intervention except in unusual circumstances.

In most cases, an on-call doctor intervened in the crises. In most patients, glycemia, peripheral oxygen saturation, and vital signs were assessed as is usually recommended,^7^ but these examinations were still not performed in many cases. Nevertheless, it is noteworthy that the level of agitation may not allow some actions to be taken. In 33% of the cases, however, no action was taken and no explanation for the crisis was pursued. Cognitive failure, namely delirium, is often disvalued by physicians and seen as inevitable in very ill and elderly patients and, therefore, not adequately investigated and managed despite its serious consequences.^8,11^

Drugs used for crisis control were benzodiazepines, antipsychotics, or both. These are the classes of drugs most used everywhere.^8,19,20^ The drug used in 12% was morphine. Morphine, although it may cause sedation as an adverse effect, is not recommended as a sedative and even considered bad practice by several guidelines.^20^ However, if pain is the suspected cause or a significant contributor to the crisis, morphine or another opioid may be a sensible choice. In these cases, pain was probably the suspected cause of agitation.

After the first episode of agitation, in only 30% of patients, there was an attempt to find a cause. Although, in most cases, the episode was not repeated, this fact can only be determined *a posteriori*. Therefore, it cannot be used as a reason for nonassessment. Agitation manifests a potentially serious condition, such as an “external sign of organ damage.”^8^ Even if it is impossible to perform an extensive assessment in the acute phase, unless the patient's state deteriorates quickly, such investigation should be performed afterward. Agitation may result from a severe condition that should be known and corrected when possible. In the notes of the physician who followed the patient, the agitation episode was recorded in only 59%, suggesting that it deserves low importance. This attitude contrasts with expert consensus on assessing and managing psychomotor agitation, recommending that agitation with no provisional diagnosis or with no available information should be presumed to be from a general medical condition until proven otherwise.^7^

This study has some weaknesses. It was performed in a limited number of internal medicine departments; therefore, the results may not be generalizable. Another potential problem was the fact that the prospective nature of the study might induce a modification in the health professionals' attitude in the departments where the study was performed. Even being a prospective study, some aspects were retrieved from the patients' files; therefore, some conclusions could have been taken from the correctness of the physicians' records and not from their reasoning, which was the objective of this study. However, even if some influence has occurred, the data obtained are still helpful and may guide future actions to improve the assessment and management of agitation in internal medicine departments.

Hopefully, this study will promote a reflection in medical departments on the attitude of health professionals, including doctors, toward assessing and managing agitated patients. Although often not approached with the deepness it deserves, agitation is a frequent problem and often the herald of a severe or even life-threatening disease that may be reverted. Coercive measures seem to be excessively frequent. Therefore, more attention should be given to this critical medical issue, including adopting guidelines on assessing and managing agitation.

## Conclusion

This study suggests that there is room for improvement regarding how agitated patients are managed. The discussion that this study may raise should include the urgent approach to the episodes of agitation, concerning assessment and control, and the use of restraints. Also, the subsequent assessment of patients to find a cause for the episode and the control of possible future crises should be re-examined. This study could be a basis for discussing this topic in internal medicine departments to write guidelines on managing agitated patients, an undoubtedly frequent and essential topic.
